# Mental Health and Social Connectedness During the COVID-19 Pandemic: An Analysis of Sports and E-Sports Players

**DOI:** 10.3389/fpsyg.2022.802653

**Published:** 2022-05-25

**Authors:** Ana Karla Silva Soares, Maria Celina Ferreira Goedert, Adriano Ferreira Vargas

**Affiliations:** Departamento de Psicologia, Universidade Federal de Mato Grosso do Sul, Campo Grande, Brazil

**Keywords:** mental health, social connectivity, sports, e-sport, wellbeing

## Abstract

Recently, the pandemic context in which the world finds itself has inspired studies that sought to evaluate to mental health and the way people are relating to the purpose of understanding and promoting improvements psychological health. The epidemiological and public health literature shows that social connection protects and promotes mental health, being an important clinical tool for reducing anxiety, depression, and stress. Thinking in the broad sense of connection, that is, feeling and perceiving oneself connected with the environment, applied to the context of sport, it is suggested that social connection could be related to the interactions in the practice of sport. Although playing sports can promote mental health, there are few findings on the topic in the context of a pandemic and with physical sports and electronic sports (e-sports) players. In this sense, the present study aims to assess the extent to which social connection and mental health indicators are correlated in a sample of sports and e-sports players. The participants were 401 Brazilian physical sports (*N* = 199, 49.6%) and e-sports players (*N* = 202, 50.4%), mostly male (53.1%) and single (59.9%), who filled in the Social Connectedness Scale (SCS), the Depression, Anxiety and Stress Scale (DASS), and demographic questions. The results indicated that social connection was negatively correlated and also predict the anxiety (*r* = −0.37), depression (*r* = −0.54), and stress (*r* = −0.39). When comparing sports and e-sports players, a statistically significant difference was identified in the levels of social connection [*t*(398) = −3.41; sports_mean_ (SD) = 4.53 (1.14); e-sports_mean_ (SD) = 4.14 (1.15)] and depression [*t*(396) = 2.90; sports_mean_ (SD) = 1.10 (0.89); e-sports_mean_ (SD) = 0.85 (0.81)]. These findings can serve as a theoretical basis for the development of intervention programs (e.g., to guide managers regarding the social distancing rules that enable them to keep holding sports practices and events) and promoting discussions that focus on the analysis of aspects promoting psychological health in sports context (physical and e-sports).

## Introduction

Throughout history, humankind has lived with pandemic situations of different proportions (e.g., black plague, Ebola, and severe acute respiratory syndrome), and in all cases, impacted in physical and psychological health were identified (Qiu et al., 2017). In March 2020, coronavirus disease 2019 (COVID-19) was declared a global pandemic by the World Health Organization (World Health Organization [WHO], 2021), and due to its high degree of infection and the fact that the virus has spread rapidly, safety measures from hygiene guidelines (e.g., use of masks and alcohol gel) to the implementation of social distancing and isolation measures were mandated.

In Brazil, different sanitary measures have been implemented (e.g., use of masks, alcohol gels, and social distancing), causing the work, educational, and inter-relational routine experienced by the population to be altered (Asmundson and Taylor, 2020), including engaging in sport due to the closure of gyms, sports centers, and public areas (Schinke et al., 2020). This aspect deserves more attention, as playing sports is considered an efficient mechanism for coping with stressful situations and promoting mental health (Tenenbaum and Eklund, 2020).

When observing the number of studies that assess aspects of mental health in the general population and in the sports context, it is observed that there is a lower prevalence of studies with a sample of sports practitioners. However, there has been an expansion of studies with sports practitioners in recent decades (Zoë et al., 2021), with findings indicating that this group is as susceptible to mental health problems as the general public (Gorczynski et al., 2017; Moesch et al., 2018). Studies in this area are fundamental, since for a long time the discussions about mental health in this group were stigmatized and those affected themselves resisted exposing the theme. Something they see changing today (Hong and Rao, 2020).

The same pattern of research that mostly considers the general population was observed in the current pandemic context, highlighting the efforts of researchers, newspapers, and scientific associations that called for studies aimed at the subset of the populace who are athletes. An example is one publication of the Association for Applied Sport Psychology (AASP), which expressed recommendations on the maintenance of mental health for athletes and practitioners of sports in this pandemic period (Byrd et al., 2020).

In this sense, recent studies have sought to fill the gap in the knowledge about the mental health status of sports practitioners and athletes during situations of pandemic and social isolation (e.g., Pillay et al., 2020; Sokić et al., 2021; Uroh and Adewunmi, 2021). Uroh and Adewunmi (2021) evaluated the psychological impact of COVID-19 social isolation measures in athletes who could not perform their activities at the time of total blockade in Nigeria. They found that individual-sport athletes experienced more psychological distress than those who played team sports. Sokić et al. (2021) explored the effects of physical activity and training routine on mental health during the COVID-19 pandemic, comparing elite vs. recreational athletes and the training routine adopted in the pandemic (inactivity, 14.7%; reduced training, 74%; and no change in the training routine, 11.3%) in a Serb sample. The results showed that elite athletes who trained less showed less anxiety than recreational athletes who also reduced training or maintained routines, suggesting that the reduction or pause in physical activities negatively impacted the mental health of the participants.

Thus, although it is important to differentiate the effects of the pandemic between different groups of athletes (Şenışık et al., 2021), athletes of different modalities (Pillay et al., 2020), and athletes with different routines (Sokić et al., 2021), it is also necessary to consider formats beyond the traditionally physical sport, such as electronic sport (e-sport). E-sport is defined as a form of sport in which the primary aspects of its practice are facilitated by electronic means, with the players, teams, and the game system mediated by a computerized interface (Hamari and Sjöblom, 2017).

The pandemic has become a propitious moment for the expansion of e-sports practice (Tudor, 2020), whose definition is not yet consolidated and involves nomenclature challenges, which often misuse the term “e-sport” (Goedert and Soares, 2020). However, scientific interest in understanding this sport in greater detail is already evident, with researchers dedicated to identifying psychosocial factors that are related to this practice (Seo and Jung, 2016).

In-depth research on the sports environment makes it possible to identify studies that seek to understand how sports activity, both physical and electronic, can be directly linked to the mental health of its players (Tang and Fox, 2016; Pluhar et al., 2019). Previous studies before the pandemic have pointed to the importance of linking physical and mental health in the practice of physical and electronic sports (Weinberg and Gould, 2017; Rudolf et al., 2020). Another important aspect related to the protection and promotion of mental health is social connection, which refers to connecting socially, be it as a group or as a person, or even as the ability to connect *per se* (Townsend and McWhirter, 2005).

In the pandemic context, studies have sought to relate aspects of social connection with psychological health, wellbeing, loneliness, and stress (Hunsaker et al., 2020; Nitschke et al., 2020; Sun et al., 2020; Wu et al., 2021). Researchers in Italy conducted an investigation on the impacts of COVID-19 on the population of the country during the lockdown period (Marotta et al., 2020), demonstrating the relevance of measuring the construct to obtain global health indicators at specific times, such as a global pandemic.

### Physical and E-Sport

When searching for the first records of any sports activity in the history of mankind, it is noted that the performance of a physical activity has always been present in human existence, but it was only in Greece that the first record of an activity focussed on competition and not only survival. The creation of the Olympic Games was the main milestone in terms of sport, and they were very close to the current vision, as they were governed by pre-established rules, relied on spectators watching, and, especially, the athletes prepared by warming up, eating a certain diet, and weight training (Tenenbaum and Eklund, 2020).

The conditions of most modern sports were established in the nineteenth century in England, bringing a standardization of pastimes with regulation and adaptation for a competition to be practiced, with modalities that emphasized different motor skills put to the test through various types of movement. Over time, other practices began to be encompassed as sports, without necessarily showing interest purely in visible bodily attributes. One example is chess, in strategizing, quick thinking, and reaction are important, traits common to other physical modalities but without requiring physical strength itself (Bottenburg, 2016).

The consideration of non-physical attributes in athletes brings to light a new concept of sport that involves the perception that there are ways to engage in a sporting activity in a completely virtual environment while maintaining certain attributes of physical sport (Kane and Spradley, 2017; Gostlin, 2021). Such competitions, called e-sports, uses electronic means, with players, teams, and the game system mediated by a computerized interface (Jonasson and Thiborg, 2010; Hamari and Sjöblom, 2017).

Research involving the topic of e-sports ends up being erroneously allocated to the online games category, since the latter encompasses a diversity of concepts that are not configured as sports. The definition of e-sport brings with it the same classic premise used to describe physical sports, as mentioned above: in short, the idea that players need to perform a competitive activity based on their skills and pre-established rules (Seo and Jung, 2016; Kane and Spradley, 2017).

### Social Connection and Mental Health

Social connection is a term widely used in the psychological field that is related both to the idea of connecting to a specific group or person and to the generalized ability to connect (Townsend and McWhirter, 2005; Malone et al., 2012; Stanley et al., 2019). It is related to the subjective recognition of being in a close relationship with people in the social world (Wu et al., 2021). The sense of connection, in general, directs an individual’s feelings as well as their thoughts and behaviors in social situations (Lee and Robbins, 1998, 2000).

Lee and Robbins (1995) consider that social connection is related to an individual’s self-opinion in relation to other people, focussing on the perceived emotional distance between themselves and friends or society. Studies point to the need to broaden the understanding of self-perception about their levels of social connection due to its important relationship with psychological health indicators, such as anxiety, depression, and stress (Williams and Galliher, 2006; Wu et al., 2021), as well as with health factors in general (Townsend and McWhirter, 2005).

According to the WHO, the presence of a social support network is a determinant of public health (Holt-Lunstad et al., 2017). Being socially connected is important to maintaining good mental health, reducing the risk of having higher anxiety, stress, and depression scores in the general population (Santini et al., 2020). Empirical evidence highlighting this relationship was observed in a longitudinal study of 21,227 New Zealanders that indicated that social connection was a stronger and more consistent predictor of mental health than the ability of mental health to predict social connection (Saeri et al., 2018).

Conversely, in the pandemic context, studies have aimed to relate social connection with mental health and its impact on the lockdown period (Marotta et al., 2020; He et al., 2021), since social isolation, especially if prolonged, can increase the risk of mental disorders (Sani et al., 2020) and increase the rates of stressors (73.4%) and depression (50.7%) and anxiety symptoms (44.7%) (Liu et al., 2020).

He et al. (2021) demonstrated that increased social connection led to an improvement in mental health indicators during COVID-19, both in the general population and among health professionals who had their mental health impacted, especially in their exposure to anxiety, depression, and lack of sleep (Cantarero et al., 2021; He et al., 2021). Wu et al. (2021) expanded these findings, highlighting that social connections with family and friends and their indirect effects on wellbeing were more potent than the effects of colleagues and neighbors, reinforcing the evidence of the relevance of social connections for wellbeing during the pandemic.

### The Present Study

In the sports context, before the pandemic, studies had evaluated the influence of sport on mental health, with the results considering the difference between different sports (Sheehan et al., 2018; Pluhar et al., 2019). Thus, they related the practice of physical activities and social connection as important variables for understanding mental health (Lamblin et al., 2017) and identified better mental health indicators in team-sport players than in individual-sport players (Doré et al., 2016; Lamblin et al., 2017). The COVID-19 pandemic also led to studies that evaluated its impact on the practice of physical activities, mental health, and social connection (Shepherd et al., 2021), with findings highlighting that in the first months of COVID-19, team-sport athletes reported more anxious and depressive symptoms than individual-sport athletes, probably because social isolation is felt more by team-sport players (McGuine et al., 2021).

As a reflection of the pandemic context, there are consistent changes in the way people related and performed activities, especially with regard to the adaptation and incorporation of new sports practices, such as e-sports. According to Kim et al. (2020), e-sport paved the way for the sports industry as a practice that involves physical and mental skills similar to other sports but practiced in a way (computer interface) that minimizes the need for physical contact and becomes a good option in a moment like the current one.

Given the above, and since the time of the pandemic generated a demand to expand the attention given to the relationship between social factors and mental health, both in the general population and in the sports context (Harandi et al., 2017), the present study aimed to evaluate the relationship between social connection and psychological health indicators in a sample of physical and electronic sport practitioners.

## Materials and Methods

### Participants and Procedure

The participants were 401 Brazilian physical sport (*N* = 199, 49.6%) and e-sport players (*N* = 202, 50.4%) who practice the sport for at least 30 min a day (physical sport—36.4%; electronic sport—43.1%) with an average age of 23 years (ranging from 18 to 57 years; *SD* = 5.14), the majority of whom were men (53.1%) and single (59.9%) without missing data. A non-probabilistic convenience sample was enrolled. To gather data, we advertised the survey link on social media (e.g., WhatsApp, Instagram, Facebook) social networks aimed at sports practitioners (e.g., university athletic groups and electronic sports players) between August and September 2020 using the snowball sampling method (Dusek et al., 2015). The primary survey screen presented information about the purpose of the survey and the voluntary and anonymous nature of its participation. As an inclusion criterion, we only considered physical and e-sport players from Brazil.

### Materials

Participants answered a set of questions about themselves (sex, age, marital status, how important is sport in your life? do you practice physical sports, electronic sports or both? on average, how much time do you dedicate to your sports practice?) and filled in the following surveys:

#### Social Connectedness Scale

This measure was originally developed by Lee and Robbins (1995) to assess the degree of interpersonal closeness experienced by individuals in different spheres (e.g., friends and society), consisting of eight items (e.g., “I feel distant from people”), answered on a six-point scale ranging from 1 (strongly agree) to 6 (strongly disagree). The original study identified adequate indicators of internal consistency (α = 0.91) and test–retest consistency over a 2-week interval (*r* = 0.96). In this study, Cronbach’s alpha and the omega score were satisfactory (α = ω = 0.88).

Depression, Anxiety and Stress Scale—Short Form (DASS-21; Lovibond and Lovibond, 1995): The scale validated in the Brazilian context (Vignola and Tucci, 2014) consists of 21 items, answered on a four-point scale ranging from 0 (does not apply to me) to 3 (applies a lot to me or most of the time). In this study, satisfactory precision indicators were identified for the factors of depression (α = ω = 0.91), stress (α = 0.85; ω = 0.87), and anxiety (α = 0.79; ω = 0.81).

### Data Analysis

For data analysis, we used PASW software (version 24). Pearson’s correlation coefficient (r) was calculated to estimate the direction and strength of the correlations between social connectedness, depression, anxiety and stress. To assess whether the correlations between the scales were significantly different between the groups of physical and electronic sports practitioners, *z*-tests were performed using the online calculator by Lenhard and Lenhard (2014). Student’s *t*-test was performed to assess player (physical and electronic) differences. Finally, we used three simple regressions to identify the predictive power of social connections on mental health (depression, stress and anxiety).

## Results

Initially, we calculated the descriptive statistics to characterize the variables of our sample. As seen in Table 1, with regard to mental health indicators, the participants showed high scores for anxiety (*M* = 1.32; *SD* = 0.79), followed by stress (*M* = 0.83; *SD* = 0.70), and depression (*M* = 0.98; *SD* = 0.86).

**TABLE 1:** Descriptive analyses of social connection and mental health.

	Total (*N* = 401)		Physical (*N* = 199)		Electronic (*N* = 202)	
	*F*	%	*F*	%	*F*	%
Female	188	46.9	120	60.3	68	33.7
Male	213	53.1	79	39.7	134	66.3
Single	240	59.9	127	63.8	113	55.9
In a relationship	122	30.4	49	24.6	73	36.1
Married	36	9.0	22	11.1	14	6.9
Divorced	2	0.5	1	0.5	1	0.5
Other	1	0.5	0	0.0	1	0.5

	**M**	**SD**	**M**	**SD**	**M**	**SD**

Age	23.4	5.14	24.50	6.35	22.49	3.31
Social connection	4.33	1.16	4.53	1.14	4.14	1.15
Mental health						
Depression	0.98	0.86	0.85	0.81	1.10	0.89
Stress	0.83	0.70	0.86	0.70	0.80	0.70
Anxiety	1.32	0.79	1.30	0.79	1.34	0.79

*M, mean; SD, standard deviation; N, sample size.*

When comparing physical and e-sports players, a statistically significant difference was identified in the levels of social connection [*t*(398) = −3.41, *p* < 0.001] and depression [*t*(396) = 2.90, *p* < 0.05], the physical sport players showing higher scores of social connection (*M* = 4.53; *SD* = 1.14) than the e-sports practitioners (*M* = 4.14; *SD* = 1.15) and the e-sports practitioners having higher levels of depression (*M* = 1.10; *SD* = 0.89) than the physical sport players (*M* = 0.85; *SD* = 0.81). A statistically significant difference was observed between the groups (physical and electronic sports) and the variables sex [χ^2^(1) = 28.56, *p* < 0.001] and age [*t*(399) = −3.99, *p* < 0.001] and there is no difference with the marital status variable [χ^2^(4) = 8.29, *p* = 0.081].

Pearson’s r was calculated to determine to what extent and direction the social connection would correlate with the mental health factors (Table 2). Social connection correlated negatively with depression (*r* = −0.54, *p* < 0.001), stress (*r* = −0.39, *p* < 0.001), and anxiety (*r* = −0.37, *p* < 0.001). There was no significant difference between physical and e-sports players in the strength of the correlation between social connection and depression (*z* = −1.108, *p* = 0.134), stress (*z* = −1.298, *p* = 0.097), or anxiety (*z* = −0.584, *p* = 0.28) (Lenhard and Lenhard, 2014).

**TABLE 2:** Correlations between social connection and mental health.

Construct	Social connection		
	Total (*N* = 401)	Physical (*N* = 199)	Electronic (*N* = 202)
Depression	−0.54*	−0.57*	−0.49*
Stress	−0.39*	−0.45*	−0.34*
Anxiety	−0.37*	−0.41*	−0.36*

*p < 0.001 (two-tailed test); mental health is represented by the variables depression, stress, and anxiety.

Next, we tested the extent to which mental health factors were predicted by social connection (Table 3). For this, three simple regression analysis considered social connection as a predictor were performed of the mental health symptoms (depression, stress, and anxiety) in total sample (Hair et al., 2015).

**TABLE 3:** Simple linear regression for mental health indicators (social connection as predictor).

	*R*	*R*^2^ Adjusted	*F*	B (SE)	*Beta*	*t*
Depression	0.54	0.29	*F*(399) = 163.67	−0.40 (0.03)	−0.54	−12.79*
Stress	0.39	0.15	*F*(399) = 71.99	−0.27 (0.03)	−0.39	−8.48*
Anxiety	0.37	0.14	*F*(399) = 63.96	−0.22 (0.03)	−0.37	−7.99*

*p < 0.001. Three simple regression analyzes were performed, one for each mental health factor.

Social connection, that is, the degree of interpersonal closeness reported by the individuals, was a negative predictor of depression (β = −0.54, *p* < 0.001), stress (β = −0.39, *p* < 0.001), and anxiety (β = −0.37, *p* < 0.001).

## Discussion

This study aimed to evaluate the relationship between social connection and mental health indicators in a sample of physical and e-sport practitioners. In addition, the presence of differences between the modalities (physical and electronic) and the predictive role of social connection with mental health indicators (depression, stress, and anxiety) was evaluated.

### Implications of Study

Our study has three main findings. First, statistically significant relationships were identified between the social connection and the mental health–describing factors, both in the total sample and by group (physical and e-sport). Second, when evaluating the difference between the modalities, a significant result was observed only in the scores of social connection and depression. Finally, social connection was a predictor of depression, anxiety, and stress levels in the total sample. Given the above, we believe that the general objective of this research has been achieved.

When we compared physical and e-sport players in the three mental health dimensions depression, anxiety, and stress, a statistically significant difference was observed only in the depression scores, the players of e-sports presenting higher depressive levels than the physical sports athletes. Thus, although physical sport has been linked to both the increase in depressive symptoms and their reduction (Reardon, 2017; Kim et al., 2020), it is important to envision this relationship in e-sports, which consists of a broadly expanding modality (Gostlin, 2021) and has advantages and disadvantages (Rasdi and Rusli, 2021). For example, digital games have been used as an intervention against depressive symptoms (Li et al., 2014) and can help reduce depression and loneliness in children (Przybylski et al., 2012). Thus, it may be that e-sport can be useful to improve the mental health of practitioners both in a normal-health context and in situations of isolation such as a pandemic that are conducive to the development of feelings of loneliness.

Regarding social connection, the findings of this study point to different ways for practitioners of the two modalities to prioritize the search for connection with other people in the group or with the environment itself (Lee et al., 2008). Practitioners of physical sport perceive themselves as more socially connected than e-sports players. The results are in line with other studies, corroborating the relevance of sport as an important tool for the promotion of psychological health, reducing levels of depression, anxiety, and stress (Townsend and McWhirter, 2005; Williams and Galliher, 2006). It should be noted that both groups’ scores were higher than the empirical median of the measure used (Lee and Robbins, 1995), suggesting that the two sports may favor the feeling of social connection and may boost mental health at a time when sports practitioners suffer from changes in their routines and are more exposed to situations conducive to depressive, stressful, and anxious states (Liu et al., 2020; McGuine et al., 2021; Shepherd et al., 2021).

Our results also highlight the relationship between social connection and mental health indicators (depression, anxiety, and stress), a negative relationship being observed, i.e., stronger perceptions of social connection were correlated with lower scores of depression, anxiety, and stress. These relationships were identified both when considering the total sample and when evaluating the modalities separately, highlighting the fact that the correlation indicators (Pearson’s r values) did not differ statistically between the groups. For this reason, a regression analysis was performed, which showed evidence of the predictive role of the social connection on the mental health of sports practitioners, both physical and electronic.

As expected, the results were in line with previous studies that highlighted the importance of promoting the social connection between practitioners of sport (Doré et al., 2016; Lamblin et al., 2017). However, little is known about this relationship when comparing physical and e-sports, since previous studies focussed more on differentiating individual and team sports (Doré et al., 2016).

Although previous studies have reported the relationship and predictive capacity of social connection with mental health indicators in the general population or with players of eminently physical sports (Saeri et al., 2018; Groarke et al., 2020; Nitschke et al., 2020), our results show that even when including computer-mediated sports in the context of COVID-19, social connection remains a relevant variable that can reduce negative psychological symptoms such as depression, stress, and anxiety. The view of social connection as a promoter of psychological health among players of both more traditional sports (physical) and a group of players who are increasingly recognized by society in general as practitioners of various forms of e-sport is broadened here.

### Limitations and Final Considerations

Despite the promising results, as with all scientific endeavors, some limitations mar this study. The first is the time when the data were collected. Restrictive measures had already been implemented due to the pandemic (e.g., social isolation and lockdown), so we could not present baseline data before the pandemic, limiting our ability to compare the levels of social connection during and before the restriction measures. In addition, due to the type of survey (online and anonymous), it was not possible to control the presence of multiple responses (despite the disclosure requesting participation only once).

A second limitation comes from the use of self-report measures. This factor could have biased the responses, since the responses given may have diverged from the true reflection of the levels of social connection, anxiety, stress, and depression. Neither physiological nor neuropsychological measures were used to corroborate the self-reported findings. Furthermore, another potential limitation convenience nature of our sample, composed by those individuals that voluntarily decided to take part in the study, which may limit the generalization of the findings. Finally, and probably the most important limitation of this study, was the cross-sectional design. We can conclude that social connection was correlated with the anxiety, stress, and depression scores, and the perception of social connection was correlated with the three mental health indicators, but these results do not imply any causal links, given the cross-sectional nature of the data.

Nevertheless, the results expand the previous observations from the literature, such as the relevance of social connections to the mental health of sports practitioners (Doré et al., 2016; Pluhar et al., 2019; McGuine et al., 2021), adding evidence about the relevance of the variables in a group of e-sports players. In addition, our findings warrant future studies in larger samples (for example, increasing the number of participants, contemplating individual and team sports in both groups, and considering elite practitioners and professionals) and studies of a longitudinal design to allow us to evaluate the evolution of the predictive role of social connection during and after the pandemic.

Although the empirical and relational character of this study and the social distancing measures used to control the COVID-19 pandemic have influenced the reduction in the social connection and practice of sport, our findings can serve as a theoretical basis for the development of intervention programs that aim, for example, to guide managers regarding the social distancing rules that enable them to keep holding sports practices and events and the maintenance of the social connection between practitioners of all modalities in pandemic situations. In addition to promoting discussions that focus on the analysis of aspects promoting psychological health not only in the practice of physical sports but also in the practice of e-sports, a modality that also promotes lower stress, depression, and negative thoughts (Kowert and Quandt, 2020) is a constant in scenarios of isolation like those required for the COVID-19 pandemic.

## Data Availability Statement

The raw data supporting the conclusions of this article will be made available by the authors, without undue reservation.

## Ethics Statement

The studies involving human participants were reviewed and approved by the Comitê de Ética em Pesquisa com Seres Humanos—Universidade Federal do Mato Grosso do SUl (CAAE: 26564719.2.0000.0021). The patients/participants provided their written informed consent to participate in this study.

## Author Contributions

AS and MG: conceptualization, study design, data analysis, and wrote the original draft. MG and AV: critical revision. AS: funding acquisition. All authors contributed to the article and approved the submitted version.

## Conflict of Interest

The authors declare that the research was conducted in the absence of any commercial or financial relationships that could be construed as a potential conflict of interest.

## Publisher’s Note

All claims expressed in this article are solely those of the authors and do not necessarily represent those of their affiliated organizations, or those of the publisher, the editors and the reviewers. Any product that may be evaluated in this article, or claim that may be made by its manufacturer, is not guaranteed or endorsed by the publisher.
